# Predicting Advanced Balance Ability and Mobility with an Instrumented Timed Up and Go Test

**DOI:** 10.3390/s20174987

**Published:** 2020-09-03

**Authors:** Ronny Bergquist, Corinna Nerz, Kristin Taraldsen, Sabato Mellone, Espen A.F. Ihlen, Beatrix Vereijken, Jorunn L. Helbostad, Clemens Becker, A. Stefanie Mikolaizak

**Affiliations:** 1 Department of Neuromedicine and Movement Science, Norwegian University of Science and Technology, 7491 Trondheim, Norway; kristin.taraldsen@ntnu.no (K.T.); espen.ihlen@ntnu.no (E.A.F.I.); beatrix.vereijken@ntnu.no (B.V.); jorunn.helbostad@ntnu.no (J.L.H.); 2 Department for Clinical Gerontology, Robert-Bosch-Hospital, 70376 Stuttgart, Germany; corinna.nerz@rbk.de (C.N.); clemens.becker@rbk.de (C.B.); s.mikolaizak@neura.edu.au (A.S.M.); 3 Department of Electrical, Electronic and Information Engineering “Guglielmo Marconi” (DEI), University of Bologna, 40136 Bologna, Italy; sabato.mellone@unibo.it

**Keywords:** iTUG, physical function, functional assessment, balance, mobility, older adults, partial least squares

## Abstract

Extensive test batteries are often needed to obtain a comprehensive picture of a person’s functional status. Many test batteries are not suitable for active and healthy adults due to ceiling effects, or require a lot of space, time, and training. The Community Balance and Mobility Scale (CBMS) is considered a gold standard for this population, but the test is complex, as well as time- and resource intensive. There is a strong need for a faster, yet sensitive and robust test of physical function in seniors. We sought to investigate whether an instrumented Timed Up and Go (iTUG) could predict the CBMS score in 60 outpatients and healthy community-dwelling seniors, where features of the iTUG were predictive, and how the prediction of CBMS with the iTUG compared to standard clinical tests. A partial least squares regression analysis was used to identify latent components explaining variation in CBMS total score. The model with iTUG features was able to predict the CBMS total score with an accuracy of 85.2% (84.9–85.5%), while standard clinical tests predicted 82.5% (82.2–82.8%) of the score. These findings suggest that a fast and easily administered iTUG could be used to predict CBMS score, providing a valuable tool for research and clinical care.

## 1. Introduction

Physical function can be measured using self-report questionnaires or supervised clinical tests that quantify the observable ability to perform tasks, such as standing up from a chair, walking, turning, or standing on a differing base of support (e.g., a single leg stance) [1]. Single tests can rarely capture multiple aspects of mobility, so a battery of tests is often administered to obtain a comprehensive picture of functional status. Many test batteries commonly used in geriatric testing and ageing research are not sensitive to change due to floor and ceiling effects [2,3], or they require considerable assessor training.

The Community Balance and Mobility Scale (CBMS) has recently been identified as a valid, reliable and comprehensive performance-based assessment for measuring physical function in seniors [2,4]. It contains a range of challenging balance and physical tasks. The test is complex, as well as time- and resource intensive. This limits its feasibility for use in large-scale public health approaches [5], or in daily clinical practice. While difficult to administer, the CBMS can be considered a more sensitive and appropriately challenging test of gait, balance and mobility as compared to other tests more commonly used in seniors [2,4,6].

There is a need for quicker, yet sensitive and robust measures to assess balance, strength, and functional decline in young and older seniors. An alternative to developing new tests is to use instrumented versions of existing validated measures. In clinical environments and ageing research, three tests are commonly used: The Short Physical Performance Battery [7], the Timed Up and Go (TUG) [8] and measure of gait speed [9]. The TUG consists of sit-to-stand transitions, walking, and turning, in one test, which is deployable and scalable. The test has been validated in most populations; it can be easily taught to health care professionals, is widely recognised, and quick to administer. The outcome measure of the TUG is the time taken to complete the whole task, measured in seconds. The ‘score’ does not discriminate fallers from non-fallers [3], identify frailty, or accurately predict falls in higher-functioning seniors [10]. Instrumenting the TUG (iTUG), using inertial sensor signals, allows measurement of spatial and temporal features from different segments of an iTUG trial, such as sit-to-stand transitions, walking, and turning. Compared to the original TUG, the iTUG has shown improved performance in assessing seniors at risk of falling, for people with Parkinson’s disease, disability, or cognitive impairment [10]. Here, we hypothesise that an iTUG performed several times back-to-back might be a comprehensive, robust, quick and feasible way to assess and extract advanced balance and mobility scores in seniors.

In order to obtain a quick and meaningful measure of functional decline in young and older seniors, we aimed to evaluate how well the averaged inertial sensor features from five iTUG repetitions could predict the CBMS total score within a group of geriatric outpatients and healthy community-dwelling seniors. Further, we sought to investigate whether the iTUG and single features of the iTUG could predict the CBMS total score accurately, compared to standard clinical tests used in routine assessments today.

## 2. Materials and Methods

### 2.1. Population

Sixty participants from two different cohorts (40 community-dwelling healthy seniors and 20 geriatric patients from an outpatient clinic) in Stuttgart, Germany, were invited to participate in this cross-sectional method study. Participants were included if they were (a) aged between 60 and 85 years and (b) able to walk 30 m independently. Exclusion criteria were any patient-reported cardiovascular, pulmonary, neurological, or mental diseases. The study was approved by the local medical ethical committee (Germany, no: 850/2018BO1), and adhered to the Declaration of Helsinki. All participants gave their written informed consent prior to inclusion.

### 2.2. Measurements

Demographic data and medical history were obtained from all participants. The following traditional non-instrumented clinical tests were completed (in order): Late Life Function and Disability Index (LLFDI) [11]; Montreal Cognitive Assessment (MoCA) [12]; Short Falls Efficacy Scale-International (FES-I) [13]; Eight-level balance scale (8-LBS) [14]; Community Balance and Mobility Scale (CBMS) [15]; 7-meter walk test (habitual and fast); 30-second chair-stand test (30-CST) [16]; Short Physical Performance Battery (SPPB) [7]; and the original TUG [8]. The iTUG measures were collected during the TUG, see Paragraph 2.4. A complete description of test administration and -outcomes can be found in Appendix A Table A1.

### 2.3. Procedures

Participants underwent assessments administered by trained research assistants (physiotherapists or sport scientists), in a hospital gait lab. The assessment battery consisted of self-reported and objectively measured tests of physical function, including the iTUG. The test order was randomised, with participants starting with either the iTUG or traditional non-instrumented clinical tests. The entire assessment battery took on average 1.5 h and participants could take breaks between tests when needed.

### 2.4. TUG and iTUG

The iTUG was performed as five consecutive repetitions of the original TUG, with 30 s break between each repetition. We used a chair with armrests that was 46 cm high. A cone was placed at a mark 3 m from the front legs of the chair. Instructions were given in accordance with those from the original TUG [8].

**Figure 1:**
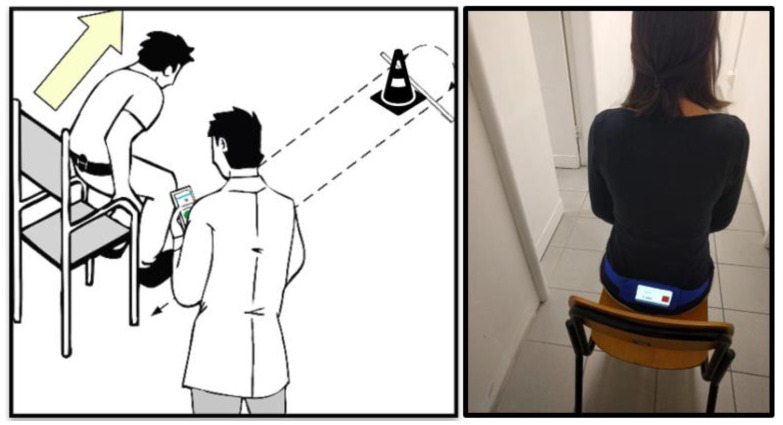
Illustration of the test set-up for the Timed Up and Go (iTUG).

During the trials the participants wore a Huawei P8 (GRA-L09) smartphone (Huawei Technologies Co, Ltd., Shenzhen, China) running a custom-made Android application, originally developed within the FARSEEING project [17]. The embedded accelerometer and gyroscope was an STMicroelectronics LSM330 (STMicroelectronics, Geneva, Switzerland), accelerometer range: +/−4 g, gyroscope range: +/−500 degrees per second (°/s), sampling rate: mean value 102.5 Hz, standard deviation 0.5 Hz, timestamp 1 nanosecond resolution resampled at 100 Hz. The smartphone was worn on their lower back in a belt case. The assessor controlled a second smartphone, which was connected via Bluetooth to the smartphone worn by the participant, to manually time each trial according to the original guidelines [8]. The sensor signals were recorded in TXT log files on the smartphone’s internal memory. The smartphone controlled by the assessor acted only as a remote controller to start and stop the recording. There was no data stream on the wireless connection and hence, no risk of data loss. The set-up is illustrated in Figure 1.

**Figure 2:**
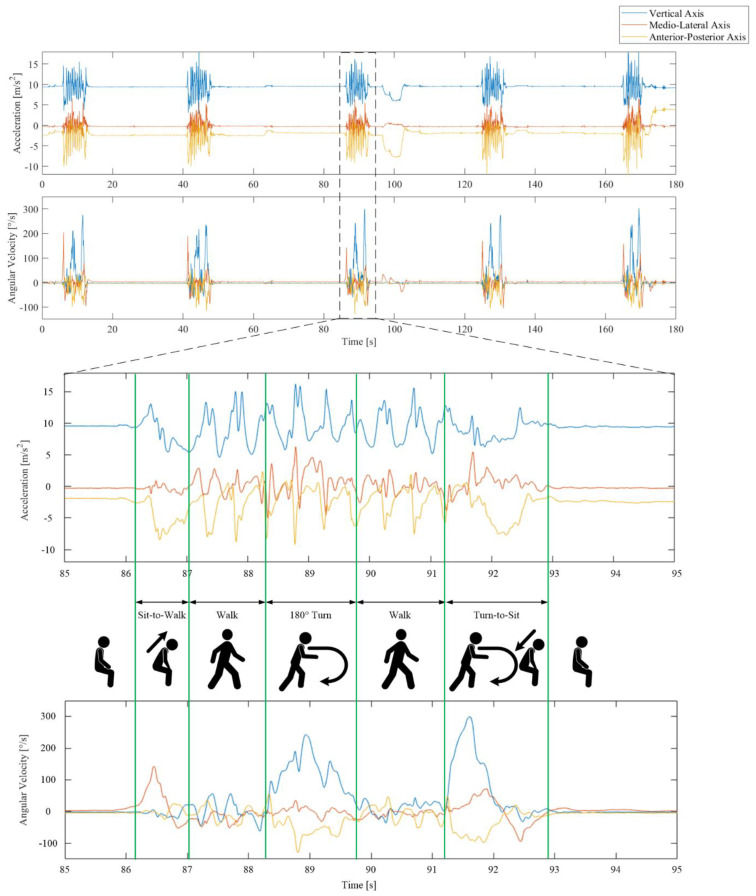
The 3-axis acceleration (upper) and angular velocity (lower) sensor signals recorded during five repetitions of an iTUG for one subject. The task is segmented into five phases separated by the green vertical lines.

Sensor signals were recorded from the triaxial gyroscope and accelerometer embedded within the smartphone worn by the participants during the iTUG. The procedures have been described elsewhere [18], but in short, we divided the iTUG into four segments: Sit-to-Walk (StW), Walk (W), First turn (FT), and Turn-to-Sit (TtS), see Figure 2. Anterior-posterior (AP) acceleration and angular velocity around the mediolateral (ML) axis were used to identify the Sit-to-Stand (StS) and walk segments. To identify the turn segments, we used the angular velocity around the vertical (V) axis. We computed 78 features from the inertial sensor signals (see Appendix A Table A2), including segment durations, intensity measured as root mean square (RMS), and the smoothness of the signal measured as normalised jerk scores (NJS). Mean and maximum angular velocities were computed from the turn segments, as well as number of steps from the walk and turn segments.

### 2.5. Statistical Analysis

**Figure 3:**
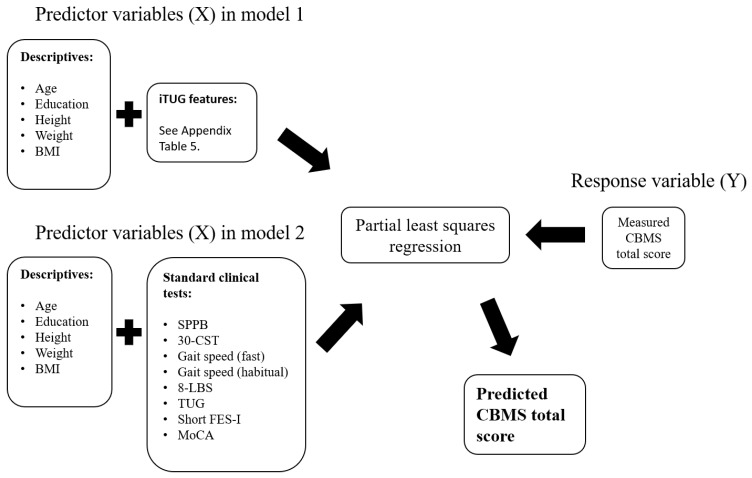
Schematic presentation of the data that were used as predictor and response variables in the two separate partial least squares regression (PLSR) models presented in this paper. Model 1 included descriptive data and iTUG features, while model two included descriptive data and standard clinical tests. The Community Balance and Mobility Scale (CBMS) scores was used as a response variable.

Demographics and other characteristics of the study population include age, sex, height, weight, body mass index (BMI), and number of years of education. Continuous variables are summarised as mean (SD). In the case of dichotomous variables, the number of participants in each category is provided. Descriptive variables were included in the partial least squares regression (PLSR) analysis in both models (Figure 3). Two PLSR analyses were run, one with iTUG features as predictor variables (predictor model 1) and one with standard clinical tests as predictor variables (predictor model 2). The response variable was the CBMS scores in both analyses.

The iTUG was performed with five repetitions to eliminate variance in performance across trials. The average value for each feature across the five repetitions was used for maximal robustness in the final model presented.

### 2.6. Partial Least Squares Regression (PLSR)

To find the variables that most accurately described the variation in CBMS total score, we used a PLSR analysis. The PLSR attempts to find the fundamental relationship between two datasets; predictor variables, X, and the response variable, Y. It combines the dimensional reduction properties known from methods such as principal component analysis (PCA) and factor analysis with regression, and identifies latent variables in the data that explain as much of the covariance as possible between the predictor variables and the response variables. Ultimately, the aim is to extract a subset of the most relevant predictors from a dataset containing many and perhaps collinear (correlated with each other) variables. The predictor variables chosen in the final model are expressed, with their corresponding loading scores (i.e., weightings), across the latent components identified in the data. The higher the loading score, the higher the relevance of that variable for that particular component of the response variable.

**Figure 4:**
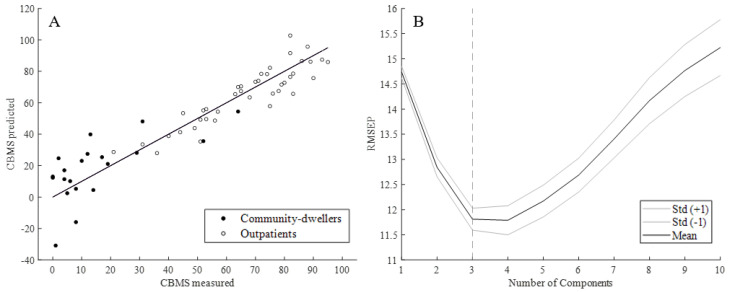
(**A**) Predicted vs. measured CBMS total score from iTUG for outpatients and community-dwellers; (**B**) Mean root mean square error of prediction (RMSEP) +/− one standard deviation across 10 components.

**Figure 5:**
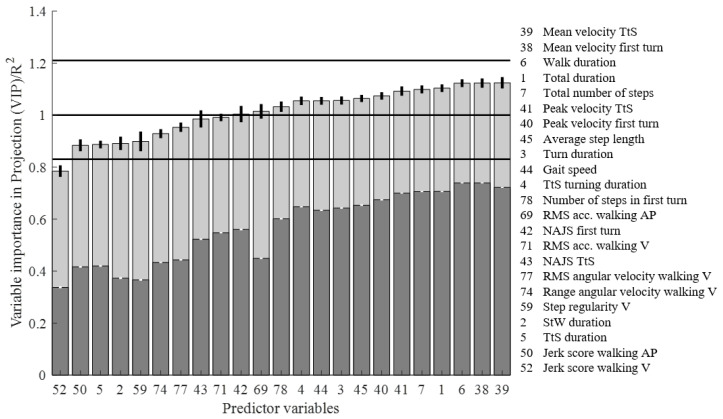
The VIP scores (light grey) and R^2^ (dark grey) of the iTUG features selected in the PLSR model. The horizontal lines represent the lower (0.83), middle (1) and upper (1.21) cut-off values used for interpreting the VIP of individual predictor variables.

The PLSR model was validated in a 7-step cross-validation procedure, to identify the most robust components without overfitting the model. We used a Monte Carlo simulation procedure with 100 repeated random iterations. X was an *n*-times-*m* matrix and Y an *n*-times-1 matrix, where *n* is the number of participants and *m* is the number of iTUG features. The X and Y data matrices were divided into six sets of matrices X’ and Y’ with equal number of rows equal to n/6. In each iteration of the Monte Carlo procedure, five of the six sets are considered training data and the last set test data. The data are repartitioned across each iteration, to identify the latent components that best explain the variation in the response variable. The iTUG features that were significantly (*p* < 0.05) cross-correlated with the training data, Xtrain and Ytrain, were selected for the PLSR model. The optimal number of components (see Figure 4B were chosen by calculating mean and standard deviation of the root mean square error of prediction (RMSEP). We compared the RMSEP between components and chose the number of components, which resulted in the minimum mean and standard deviation of RMSEP. We then calculated the variable importance in projection (VIP), which is an accumulated measure of the importance of each variable from each component in the PLSR model. The most common VIP cut-off for variable selection is a VIP value of >1, but variables with VIP values between 0.83 and 1.21 are also used in some situations [19], hence we chose to illustrate how the variables selected in our PLSR method align with all these three cut-offs (Figure 5). Finally, Z-scores were obtained to analyse the statistical difference in RMSEP between the iTUG model and the model of standard clinical tests. Alpha was set to 0.05. All computations were performed in MATLAB 2019b (MathWorks, Natick, MA, USA).

## 3. Results

Sixty participants were included in the study (mean age 74.2 years ± 7.6), 32 females (53.3%)). The characteristics and scores of physical function are presented in Table 1.

### 3.1. PLSR of iTUG Features versus the CBMS

Using the PLSR model with iTUG-features as predictors, and CBMS total score as the response variable, we found that the first three components of iTUG features predicted the CBMS total score with an regression coefficient (R^2^) of 0.852 (95% CI 0.849–0.855, see Table 2).

The RMSEP (see Figure 4) was found to be lowest with 3 and 4 components, where 4 was slightly lower than 3 (11.79 vs. 11.81), albeit not significantly so (*p* = 0.9).

### 3.2. PLSR of Standard Clinical Tests vs. the CBMS

In the PLSR model, with standard clinical test scores as predictors and CBMS total score as the response variable, we found that the first two components predicted the CBMS total score with an R^2^ of 0.825 (95% CI 0.822–0.828, see Table 3).

**Figure 6:**
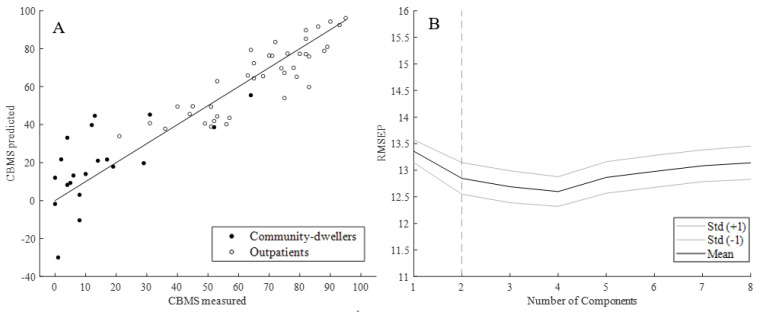
(**A**) Predicted vs. measured CBMS total score from standard clinical tests for outpatients and community-dwellers; (**B**) mean RMSEP +/− one standard deviation across eight components.

**Figure 7:**
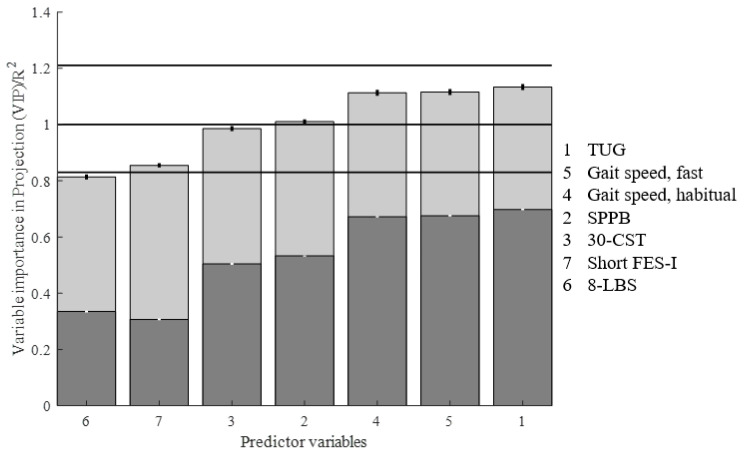
The VIP scores (light grey) and R^2^ (dark grey) of the clinical variables selected in the PLSR model. The horizontal lines represent the lower (0.83), middle (1), and upper (1.21) cut-off values used for interpreting the VIP of individual predictor variables.

Mean RMSEP with two components was 12.85 (Figure 6). The RMSEP was not significantly lower with more than two components, and RMSEP of CBMS prediction with the iTUG model was significantly lower than with standard clinical tests (Figure 4B and Figure 6B, *p* ≤ 0.0001). Four variables in the model had VIP values between the cut-offs 1 and 1.2, two were between 0.8 and 1, and one variable had a VIP below 0.8 (Figure 7). 

## 4. Discussion

This study aimed to assess whether signal features averaged from five iTUG trials could predict CBMS scores as a ground truth model in community-dwelling seniors and geriatric outpatients, using a PLSR analysis. In addition, this study sought to investigate whether the predictive ability of iTUG was superior to standard clinical tests in predicting CBMS scores.

The PLSR model of iTUG features predicted the CBMS score with a substantial level of predictive accuracy (mean explained variation of 85.2%) [20]. The iTUG model had a similar predictive ability of CBMS scores as a battery of clinical tests, and significantly less error of prediction.

CBMS evaluates high-level gait, balance and mobility, required for safe and independent living in the community [4,6]. Our findings suggest that a five-time repeated iTUG, which requires little floor space, a smartphone, and about 5 min in the clinic or lab, can accurately predict a person’s score on the CBMS, which otherwise would require 20–25 min, larger facilities and resources to administer. While the standard clinical tests were also able to predict the CBMS score with high accuracy, the test battery took approximately 35 min to administer, and with staff needing specific training to be able to collect those data. For iTUG, testing can be completed within five minutes and minimal training is required to administer the test.

The signal features with highest loading scores on the first component are features that represent several different segments of the iTUG, including walking, turning and turn-to-sit. We also found that they represent different units, such as velocity, duration, number of steps, and step length. These findings indicate that no specific signal features stand out from the others in terms of how much of the variation in CBMS they describe, but rather that a good prediction of CBMS relies on several complementary pieces of information. However, six of the ten features with highest R^2^ scores were features obtained from the two turning phases of the iTUG, perhaps not coincidental, as the importance of turning for predicting balance have been recognised in several other studies. For example, an earlier study on older adults found that those who had poorer scores on the Berg Balance Scale and the Fullerton Advanced Balance scale, exhibited slower turns in the iTUG [21]. In a study of high-functioning young seniors, the features ‘Walk duration’ and ‘TtS maximum velocity’ both had significant discriminative ability on self-reported physical function as measured by the LLFDI [18]. Turning features of the iTUG have also been found to be sensitive for testing people with impaired motor control due to neurological conditions [22,23,24], fallers [25], and in persons with mild cognitive impairment [26], which could also be explained by motor control impairment [26,27].

### 4.1. Limitations

We acknowledge that this study has some limitations. First, the sample size was relatively small. It is generally recognised that machine learning-based prediction models trained on small sample-sizes are vulnerable to biased performance estimates [28]. This study was intended as a pilot study, and a larger study with additional or larger cohorts is necessary to confirm the findings. In addition, the model described here has not been validated on an external dataset, where the same procedures have been applied. Therefore, the presented results need to be interpreted with caution and cannot be generalised to other populations without further confirmation of this work.

### 4.2. Implications for Clinical Practice and Future Research

We found that five trials of iTUG, which require very little time, space and training to administer, could predict the CBMS with a substantial and slightly higher accuracy than a battery of standard clinical tests. The potential implications of these findings are that the use of instrumented tests would save time for the individual and clinicians, thus avoiding patient fatigue associated with comprehensive test batteries [5]. Furthermore, self-administrable iTUGs are on the rise, which would allow seniors to assess their ability from the comfort of their own home [29]. The adoption of electronic technologies has been recognised as a key strategy for cutting costs in healthcare [30]. With the iTUG, healthy seniors as well as patients could use their own smartphone, with their physical function monitored remotely by clinicians or researchers.

In future work, the iTUG PLSR model should be trained on a larger dataset and validated externally in new data collected using the same procedures as the training data. The same procedure as used in the current analysis could also be applied to evaluate how well the iTUG can predict relevant outcomes for other populations with impaired physical function, such as Parkinson’s disease, multiple sclerosis, chronic obstructive pulmonary disease, stroke, and others.

## 5. Conclusions

In this study, we demonstrated that averaged signal features from a smartphone worn during a 5-times repeated iTUG could predict the CBMS score in community-dwellers and outpatients with 85.2% accuracy, while more elaborate standard clinical tests could predict it with 82.5% accuracy. The results suggest that an iTUG, which is potentially cost-saving, fast, and easy to administer, may be used to predict a person’s score on the CBMS in face-to-face and remotely conducted research and clinical care.

## Figures and Tables

**Table 1:** Participants’ characteristics. Mean and standard deviation (SD) for all variables except sex distribution.

	Community-Dwellers (n = 40)	Outpatients (n = 20)
Age, in years	71.8 (7.3)	78.9 (5.9)
Sex (M/F)	17/23	11/9
Years of education	14.6 (3.7)	11.5 (4.3)
Height (cm)	169.5 (9.6)	165.9 (11.1)
Weight (kg)	72 (12.6)	75.5 (16.9)
BMI (kg/m^2^)	25.0 (3.6)	27.3 (4.3)
MoCA (0–30)	27.1 (4.9)	23.7 (2.2)
CBMS (0–96)	66.7 (18.3)	15.0 (17.2)
LLFDI (0–100)	75.5 (9.9)	51.3 (14.4)
TUG (s)	8.3 (1.2)	13.9 (4.0)
SPPB (0–12)	11.7 (0.9)	9.0 (2.4)
8-LBS (1–8)	5.4 (1.5)	4.3 (1.4)
30-CST (no. of repetitions)	15.3 (2.9)	8.8 (3.3)
Gait speed, habitual (m/s)	1.36 (0.20)	0.88 (0.21)
Gait speed, fast (m/s)	1.83 (0.27)	1.18 (0.30)
Short FES-I (7–28)	8.1 (1.5)	10.7 (4.0)

M, male; F, female; BMI, body mass index; MoCA, Montreal Cognitive Assessment; CBMS, Community Balance and Mobility Scale; LLFDI, Late Life Function and Disability Index; TUG, Timed Up and Go; SPPB, Short Physical Performance Battery; 8-LBS, Eight Level Balance Scale; 30-CST, 30-s Chair Stand; FES-I, Falls Efficacy Scale-International.

**Table 2:** Loading scores, regression coefficient (R^2^) and variable importance in projection (VIP) scores for all variables selected, and R^2^ of the first three components in the PLSR analysis for iTUG features and descriptive variables.

Variables Selected by PLSR	Loading Scores, Component 1	Loading Scores, Component 2	Loading Scores, Component 3	VIP	R^2^
**iTUG features**					
Mean velocity first turn [°/s]	0.215	0.032	−0.091	1.123	0.739
Walk duration [s]	−0.222	0.024	0.028	1.123	0.739
Mean velocity TtS [°/s]	0.207	0.094	−0.069	1.124	0.722
Total duration [s]	−0.218	0.030	0.015	1.104	0.706
Total number of steps	−0.213	−0.039	0.079	1.099	0.706
Peak velocity TtS [°/s]	0.209	0.045	−0.035	1.092	0.700
Peak velocity first turn [°/s]	0.207	−0.001	−0.056	1.074	0.674
Average step length [m]	0.204	0.067	−0.054	1.064	0.654
TtS turning duration [s]	−0.205	−0.032	0.065	1.055	0.647
Turn duration [s]	−0.208	0.000	0.130	1.056	0.643
Gait speed [m/s]	0.207	−0.009	0.009	1.055	0.634
Number of steps in first turn	−0.201	−0.020	0.200	1.032	0.601
NAJS first turn	−0.182	−0.121	0.205	1.004	0.560
RMS acc. walking V [m/s^2^]	0.196	−0.112	0.074	0.991	0.548
NAJS TtS	−0.173	−0.177	0.130	0.985	0.523
RMS acc. walking AP [m/s^2^]	0.191	−0.266	0.080	1.014	0.449
RMS angular velocity walking V [°/s^2^]	0.185	−0.188	0.012	0.953	0.443
Range angular velocity walking V [°/s^2^]	0.181	−0.182	0.042	0.929	0.433
TtS duration [s]	−0.173	0.061	0.091	0.887	0.419
Jerk score walking AP [m^−1^]	0.174	−0.137	−0.048	0.884	0.416
StW duration [s]	−0.140	−0.076	−0.203	0.891	0.373
Step regularity V [%]	0.138	0.163	−0.070	0.899	0.366
Jerk score walking V	0.143	0.073	−0.047	0.785	0.338
**Descriptives**					
Age	−0.129	−0.401	−0.327	1.404	0.472
Education	0.113	0.496	0.047	1.188	0.352
	Component 1	Component 2	Component 3		Total
Mean Explained Variation (R^2^)	0.771	0.058	0.023		0.852
95% CI	0.769–0.772	0.054–0.061	0.020–0.027		0.849–0.855

ACRONYMS: TtS: Turn to Sit; NAJS: Normalised Angular Jerk Score; RMS: Root Mean Square; V: Vertical.

**Table 3:** Loading scores, R^2^ and VIP scores for all variables selected and R^2^ of the first two components in the PLSR analysis for standard clinical tests and descriptive variables.

Variables Selected by PLSR	Loading Scores, Component 1	Loading Scores, Component 2	VIP	R^2^
**Clinical**				
TUG	−0.398	0.102	1.133	0.698
Gait speed, fast	0.385	−0.057	1.115	0.676
Gait speed, habitual	0.381	−0.034	1.113	0.672
SPPB	0.371	−0.260	1.010	0.533
30-CST	0.346	−0.320	0.985	0.504
8-LBS	0.257	0.215	0.814	0.335
Short FES-I	−0.314	0.451	0.855	0.307
**Descriptives**				
Age	−0.250	−0.728	1.065	0.472
Education	0.270	0.331	0.843	0.352
	Component 1	Component 2		Total
Mean explained variation (R^2^)	0.798	0.027		0.825
95% CI	0.796–0.801	0.024–0.029		0.822–0.828

## Appendix A

**Table A1:** Complete list of standard clinical tests with description.

**Late Life Function and Disability Index (LLFDI)** The LLFDI [11] consists of two parts, function and disability, with 32 and 16 items, respectively. Both parts of the LLFDI were administered, but for the purpose of this study, only the functional scores will be used in the analysis. The items are regarding how much difficulty the participant experiences with carrying out different activities of daily life with a rating scale of 1–5, ranging from no difficulty to cannot do. The questions span across three dimensions; upper extremity, lower extremity, and advanced lower extremity. The total score is scaled, resulting in scores ranging from 0–100 (higher score indicating better performance), allowing comparison to other trials and cohorts. **Short Falls-Efficacy Scale International (Short FES-I)** The Short FES-I (9) is a 10-item questionnaire developed to assess the fear of falling in community-dwelling older adults. The outcome is a sum score ranging from minimum 7 (no concern about falling) to maximum 28 (severe concern about falling). **Montreal Cognitive Assessment (MoCA)** MoCA [12] is a screening tool used identify mild cognitive impairment (MCI) that assesses short-term memory, visuospatial abilities, executive functions, attention, concentration and working memory, language, and orientation to time and place. The score ranges from 0 to a maximal score of 30, with a higher score indicating better cognitive function. **Eight-Level Balance Scale (8-LBS)** The 8-LBS (11) is a test of static balance in which the participants attempt increasingly difficult positions for 15 s. The test ends when (if) the participants are not able to hold the position for 15 s. The positions are (1) side-by-side standing, narrow base, eyes open; (2) side-by-side standing, narrow base, eyes closed; (3) semi-tandem, eyes open; (4) tandem, eyes open; (5) tandem, eyes closed; (6) one-leg standing, eyes open; (7) one-leg standing, eyes closed; (8) one-leg stand, eyes closed + cognitive distraction (mentioning of the months of the year in a backwards order). The outcome is the number (in order) of the most difficult position attempted, ranging from 1 (least difficult) to 8 (most difficult). **Community Balance and Mobility Scale (CBMS)** The CBMS (14) is a test battery of balance and mobility consisting of 13 tasks, of which six are assessed unilaterally. It has been shown to be a promising performance-based test of physical function in higher-functioning seniors (2, 6). Each task is rated at the assessor’s discretion, and a score given from 0 (unable) to 5 (coordinated and controlled, without excessive equilibrium reactions). The scores are summed and the total score ranges from 0 to 96, where a higher score indicates better performance. The bonus point (95 + 1) is given if the participants can descend a staircase while holding a weighted basket in front of them, allowed only intermittently to look at the steps. **7-Meter Walk Test (Habitual and Fast)** Participants are timed over 7 m within a 9-meter track, allowing one meter in each end for acceleration/deceleration. The best time from two trials in both habitual and fast walking conditions were used to calculate respective gait speeds (m/s). **30-Second Chair-Stand Test (30-CST)** In the 30-CST the assessor counts the number of repetitions of sit-to-stands the participants can perform in 30 s. The test was developed to overcome the floor-effect associated with the Five times sit-to-stand, and is originally a part of the Fullerton Functional Fitness Test battery (12). **Short Physical Performance Battery (SPPB)** SPPB is a test of physical functioning of the lower extremities in older adults (13). The test consists of three parts, where the participants (1) attempts to keep their balance in three different feet-positions for 10 s in each, (2) walk four meters in habitual pace (performed twice), and (3) perform five repeated sit-to-stands as fast as possible. Each part is scored, and a combined score from 0–12 is given, where a higher score indicates better performance.

**Table A2:** Complete list of extracted iTUG features used in the PLSR analysis.

Total Duration	RMS ML gyro StW	Peak Velocity TtS	Stride Regularity ML
StW Duration	RMS V gyro StW	NAJS 180° Turn	Stride Regularity V
180° Turn Duration	Range AP acc TtS	NAJS TtS	Gait Symmetry AP
TtS Turning Duration	Range ML acc TtS	Gait Speed	Gait Symmetry ML
TtS Duration	Range V acc TtS	Average Step Length	Gait Symmetry V
Walk Duration	RMS AP acc TtS	Step Duration	Range Acceleration Walking AP
Total Number of Steps	RMS ML acc TtS	Standard Dev. of Step Duration	Range Acceleration Walking ML
Range AP acc StW	RMS V acc TtS	Coef. Variation of Step Duration	Range Acceleration Walking V
Range ML acc StW	Jerk Score AP TtS	Coordination Index	RMS Acceleration Walking AP
Range V acc StW	Jerk Score ML TtS	Jerk Score Walking AP	RMS Acceleration Walking ML
RMS AP acc StW	Jerk Score V TtS	Jerk Score Walking ML	RMS Acceleration Walking V
RMS ML acc StW	Range AP gyro TtS	Jerk Score Walking V	Range Angular Velocity Walking AP
RMS V acc StW	Range ML gyro TtS	Normalised Jerk Score Walking AP	Range Angular Velocity Walking ML
Jerk Score AP StW	Range V gyro TtS	Harmonic Ration AP	Range Angular Velocity Walking V
Jerk Score ML StW	RMS AP gyro TtS	Harmonic Ration ML	RMS Angular Velocity Walking AP
Jerk Score V StW	RMS ML gyro TtS	Harmonic Ration V	RMS Angular Velocity Walking ML
Range AP gyro StW	RMS V gyro TtS	Step Regularity AP	RMS Angular Velocity Walking V
Range ML gyro StW	Mean Velocity 180° Turn	Step Regularity ML	Number of Steps in 180° Turn
Range V gyro StW	Mean Velocity TtS	Step Regularity V	
RMS AP gyro StW	Peak Velocity 180° Turn	Stride Regularity AP	

ACRONYMS: ML: Medio-Lateral; acc: acceleration; V: Vertical; Coef: Coefficient.

## References

[1] Freiberger E., De Vreede P., Schoene D., Rydwik E., Mueller V., Frändin K., Hopman-Rock M. (2012). Performance-based physical function in older community-dwelling persons: A systematic review of instruments. Age Ageing.

[2] Bergquist R., Weber M., Schwenk M., Ulseth S., Helbostad J.L., Vereijken B., Taraldsen K. (2019). Performance-based clinical tests of balance and muscle strength used in young seniors: A systematic literature review. BMC Geriatr..

[3] Schoene D., Wu S.M., Mikolaizak A.S., Menant J.C., Smith S.T., Delbaere K., Lord S.R. (2013). Discriminative ability and predictive validity of the timed up and go test in identifying older people who fall: Systematic review and meta-analysis. J. Am. Geriatr. Soc..

[4] Weber M., Van Ancum J., Bergquist R., Taraldsen K., Gordt K., Mikolaizak A.S., Nerz C., Pijnappels M., Jonkman N.H., Maier A.B. (2018). Concurrent validity and reliability of the Community Balance and Mobility scale in young-older adults. BMC Geriatr..

[5] Gordt K., Mikolaizak A.S., Taraldsen K., Bergquist R., Van Ancum J.M., Nerz C., Pijnappels M., Maier A.B., Helbostad J.L., Vereijken B. (2020). Creating and Validating a Shortened Version of the Community Balance and Mobility Scale for Application in People Who Are 61 to 70 Years of Age. Phys. Ther..

[6] Balasubramanian C.K. (2015). The community balance and mobility scale alleviates the ceiling effects observed in the currently used gait and balance assessments for the community-dwelling older adults. J. Geriatr. Phys. Ther..

[7] Guralnik J.M., Simonsick E.M., Ferrucci L., Glynn R.J., Berkman L.F., Blazer D.G., Scherr P.A., Wallace R.B. (1994). A short physical performance battery assessing lower extremity function: Association with self-reported disability and prediction of mortality and nursing home admission. J. Gerontol..

[8] Podsiadlo D., Richardson S. (1991). The timed “Up & Go”: A test of basic functional mobility for frail elderly persons. J. Am. Geriatr. Soc..

[9] Studenski S., Perera S., Patel K., Rosano C., Faulkner K., Inzitari M., Brach J., Chandler J., Cawthon P., Connor E.B. (2011). Gait Speed and Survival in Older Adults. JAMA.

[10] Sprint G., Cook D.J., Weeks D.L. (2015). Toward Automating Clinical Assessments: A Survey of the Timed Up and Go. IEEE Rev. Biomed. Eng..

[11] Haley S.M., Jette A.M., Coster W.J., Kooyoomjian J.T., Levenson S., Heeren T., Ashba J. (2002). Late Life Function and Disability Instrument: II. Development and evaluation of the function component. J. Gerontol. A Biol. Sci. Med. Sci..

[12] Nasreddine Z.S., Phillips N.A., Bédirian V., Charbonneau S., Whitehead V., Collin I., Cummings J.L., Chertkow H. (2005). The Montreal Cognitive Assessment, MoCA: A brief screening tool for mild cognitive impairment. J. Am. Geriatr. Soc..

[13] Kempen G.I., Yardley L., van Haastregt J.C., Zijlstra G.A., Beyer N., Hauer K., Todd C. (2008). The Short FES-I: A shortened version of the falls efficacy scale-international to assess fear of falling. Age Ageing.

[14] Clemson L., Fiatarone Singh M.A., Bundy A., Cumming R.G., Manollaras K., O’Loughlin P., Black D. (2012). Integration of balance and strength training into daily life activity to reduce rate of falls in older people (the LiFE study): Randomised parallel trial. BMJ.

[15] Howe J.A., Inness E.L., Venturini A., Williams J.I., Verrier M.C. (2006). The Community Balance and Mobility Scale--a balance measure for individuals with traumatic brain injury. Clin. Rehabil..

[16] Rikli R.E., Jones C.J. (1999). Development and validation of a functional fitness test for community-residing older adults. J. Aging Phys. Act..

[17] Mellone S., Tacconi C., Schwickert L., Klenk J., Becker C., Chiari L. (2012). Smartphone-based solutions for fall detection and prevention: The FARSEEING approach. Z. Gerontol. Geriatr..

[18] Coni A., Van Ancum J.M., Bergquist R., Mikolaizak A.S., Mellone S., Chiari L., Maier A.B., Pijnappels M. (2019). Comparison of Standard Clinical and Instrumented Physical Performance Tests in Discriminating Functional Status of High-Functioning People Aged 61–70 Years Old. Sensors.

[19] Mehmood T., Liland K.H., Snipen L., Sæbø S. (2012). A review of variable selection methods in Partial Least Squares Regression. Chemom. Intell. Lab. Syst..

[20] Hair J.F., Ringle C.M., Sarstedt M. (2011). PLS-SEM: Indeed a silver bullet. J. Mark. Theory Pract..

[21] Chen T., Chou L.-S. (2017). Effects of Muscle Strength and Balance Control on Sit-to-Walk and Turn Durations in the Timed Up and Go Test. Arch. Phys. Med. Rehabil..

[22] Caronni A., Sterpi I., Antoniotti P., Aristidou E., Nicolaci F., Picardi M., Pintavalle G., Redaelli V., Achille G., Sciumè L. (2018). Criterion validity of the instrumented Timed Up and Go test: A partial least square regression study. Gait Posture.

[23] Palmerini L., Mellone S., Avanzolini G., Valzania F., Chiari L. (2013). Quantification of motor impairment in Parkinson’s disease using an instrumented timed up and go test. IEEE Trans. Neural Syst. Rehabil. Eng..

[24] Salarian A., Horak F.B., Zampieri C., Carlson-Kuhta P., Nutt J.G., Aminian K. (2010). iTUG, a sensitive and reliable measure of mobility. IEEE Trans. Neural Syst. Rehabil. Eng..

[25] Greene B.R., O’Donovan A., Romero-Ortuno R., Cogan L., Scanaill C.N., Kenny R.A. (2010). Quantitative Falls Risk Assessment Using the Timed Up and Go Test. IEEE Trans. Biomed. Eng..

[26] Mirelman A., Weiss A., Buchman A.S., Bennett D.A., Giladi N., Hausdorff J.M. (2014). Association between performance on Timed Up and Go subtasks and mild cognitive impairment: Further insights into the links between cognitive and motor function. J. Am. Geriatr. Soc..

[27] Buchman A.S., Boyle P.A., Leurgans S.E., Barnes L.L., Bennett D.A. (2011). Cognitive function is associated with the development of mobility impairments in community-dwelling elders. Am. J. Geriatr. Psychiatry.

[28] Vabalas A., Gowen E., Poliakoff E., Casson A.J. (2019). Machine learning algorithm validation with a limited sample size. PLoS ONE.

[29] Bergquist R., Vereijken B., Mellone S., Corzani M., Helbostad J.L., Taraldsen K. (2020). App-based Self-administrable Clinical Tests of Physical Function: Development and Usability Study. JMIR Mhealth Uhealth.

[30] Kvedar J., Coye M.J., Everett W. (2014). Connected Health: A Review Of Technologies And Strategies To Improve Patient Care With Telemedicine And Telehealth. Health Aff..

